# How much is enough? Defining the critical resection margin in myxofibrosarcoma

**DOI:** 10.1186/s12957-026-04403-6

**Published:** 2026-05-11

**Authors:** Christoph Wallner, Marius Drysch, Felix Reinkemeier, Sonja Schmidt, Alexander Sogorski, Ingo Stricker, Mehran Dadras, Marcus Lehnhardt, Flemming Puscz

**Affiliations:** 1 Clinic for Plastic and Reconstructive Surgery, BG University Hospital Bergmannsheil Bochum, Ruhr- University Bochum, Bürkle-de-la-Camp-Platz 1, Bochum, 44789 Germany; 2 Institute of Pathology, Ruhr-University Bochum, Bochum, Germany; 3 Clinic for Plastic and Reconstructive Surgery, Agaplesion Diakonieklinikum Hamburg, Hamburg, Germany

**Keywords:** Myxofibrosarcoma, Surgical margins, Local recurrence, Radiotherapy, Exploratory modeling, Risk assessment

## Abstract

**Background:**

Myxofibrosarcoma (MFS) is characterized by infiltrative growth and high local recurrence (LR) rates. Despite consensus on the importance of negative resection margins (R0), the prognostic significance of quantitative margin width beyond R0 status remains unclear.

**Methods:**

This single-center retrospective study included 94 patients with histologically confirmed myxofibrosarcoma treated between 2000 and 2023. Resection margins, recurrence patterns, and treatment variables were analyzed using descriptive stratification, exploratory threshold scanning with multiplicity correction, and complementary computational approaches for internal signal exploration.

**Results:**

Local recurrence occurred in 28/84 patients (33%; 28/79, 35% among those with documented recurrence status). Numeric margin width was available in 38/84 cases (45%). Exploratory threshold analyses showed the smallest unadjusted signal near 0.3 mm, but this finding did not remain consistently significant after multiplicity correction. Radiotherapy was associated with numerically lower recurrence rates, although subgroup comparisons remained non-significant and vulnerable to confounding by indication. Complementary computational analyses yielded overlapping margin-related signals, but all such findings were interpreted as exploratory given the limited sample size, sparse events, and lack of external validation.

**Conclusions:**

In this exploratory analysis, very close resection margins may define a higher-risk zone for local recurrence, but the observed signal near 0.3 mm should not be interpreted as a clinically established threshold. R0 status (“tumor not on ink”) and anatomical barrier quality remain the primary determinants of margin adequacy in myxofibrosarcoma. Prospective multicenter validation with standardized pathology and harmonized time-to-event data is required.

## Introduction

Myxofibrosarcoma (MFS) represents one of the most common adult soft tissue sarcomas (STS), characterized by an infiltrative growth pattern, high local recurrence (LR) rates, and considerable histopathological heterogeneity. Its hallmark myxoid stroma and “tail sign” extensions observed on imaging contribute to its elusive boundaries and complicate surgical management [1–3].

Surgical excision with negative margins (R0) remains the cornerstone of curative therapy. However, unlike other STS subtypes, MFS displays a heightened sensitivity to subtle variations in margin width. Recent cohort studies have highlighted that quantitative thresholds, particularly those at or above 1–2 mm, carry prognostic relevance for local control. Yurtbay et al. demonstrated that margins > 1 mm were associated with a 2-year LRFS of 91.7%, compared to 46.9% for ≤ 1 mm [4]. Similarly, an Australian cohort found a 2 cm cutoff to be predictive of improved local recurrence-free survival (LRFS) and overall survival (OS) (HR 0.22 and 0.23, respectively) [5]. Nevertheless, data from the Vienna and Czech groups suggest that margin quality (i.e. R-status) may supersede width once R0 is achieved, thus shifting emphasis toward histologically negative boundaries over arbitrary distance alone [3, 6].

The role of adjuvant radiotherapy (RT) is increasingly recognized as pivotal in the multidisciplinary management of MFS. Radiotherapy not only enhances local control following close or positive margins, but also may compensate for anatomical limitations that preclude wide excision. In a multicenter French cohort of 425 patients, adjuvant RT improved 5-year LRFS across both R0 and R1 strata (from ~ 70% to ~ 77% and ~ 50% to ~ 75%, respectively), underscoring its additive benefit [7, 8].

Despite advances in sarcoma management, a standardized margin threshold specific to MFS remains elusive. Current consensus statements (e.g. NCCN, ESMO) emphasize individualized resection strategies, accounting for histologic subtype, critical structures, and multimodal therapy integration [9, 10]. Moreover, the advent of machine learning and nomogram-based modeling has begun to augment traditional statistical tools, offering individualized recurrence risk stratification and dynamic cutoff optimization. However, most published models remain limited by lack of granular margin data and an overemphasis on overall survival endpoints [11, 12].

This study aimed to explore the association between resection margin width and local recurrence in a single-center MFS cohort using stratified descriptive analyses, exploratory threshold scanning with multiplicity control, and complementary computational approaches. We sought to characterize the direction, magnitude, and uncertainty of any margin–recurrence signal rather than to define a definitive clinically applicable threshold.

## Methods

### Study design and ethical approval

This retrospective, single-center cohort study was conducted in accordance with the Declaration of Helsinki and approved by the local ethics committee of the Ruhr-University Bochum (approval number 20–7126). Written informed consent for data collection and analysis was obtained from all patients.

### Patient cohort

The study included all consecutive adult patients diagnosed with MFS who underwent surgical treatment at a certified sarcoma center between January 2000 and December 2023. The diagnosis was confirmed by experienced sarcoma pathologists in accordance with the WHO classification. All histological reports were reviewed within a multidisciplinary tumor board. MFS cases were identified from an institutional sarcoma registry (*n* = 1,060 total entries), yielding a union cohort of 84 patients after formal selection audit.

As a tertiary referral center, the institution receives a substantial proportion of patients after external primary surgery or with positive margins requiring re-resection. An external/re-resection flag was constructed from registry fields and cross-validated against documented R-status for sensitivity analyses. Classification remained uncertain for 37/84 patients (44.0%) where both source fields were undocumented.

### Pathological margin assessment

The minimum resection margin was measured as the shortest tumor-to-ink distance on formalin-fixed, paraffin-embedded specimens after standardized inking of resection surfaces. R0 status was defined as no tumor cells at the inked margin; R1 indicated microscopic tumor at ink. Because margin assessment was based on formalin-fixed, inked specimens, submillimeter values are inherently vulnerable to fixation-related shrinkage and sectioning or inking artefacts. Infiltrative extensions characteristic of myxofibrosarcoma were not systematically mapped in the present retrospective cohort.

### Variable harmonization and missingness

Clinicopathological variables were harmonized from the registry: minimum resection margin (continuous and categorized as < 1 mm, 1–5 mm, > 5 mm, or missing), tumor grade (low: G1–2; high: G3), maximum tumor dimension, tumor depth, anatomical location, radiotherapy status (neoadjuvant and adjuvant RT could not be reliably distinguished), and local recurrence (binary). Missingness was reported for all variables. Radiotherapy status was harmonized from registry variables capturing perioperative radiotherapy exposure. However, neoadjuvant and adjuvant treatment could not always be reliably distinguished. Radiotherapy-related findings were therefore interpreted primarily as descriptive and potentially confounded by indication.

### Machine learning and statistical analyses

Baseline characteristics are presented as medians with IQR for continuous variables and counts with percentages for categorical variables. An exploratory threshold scan was performed using Fisher exact tests across candidate cutoffs meeting a minimum group size requirement, with multiplicity adjustment by Bonferroni correction and permutation-based family-wise error control (5,000 permutations, minimum-p approach).

To complement the descriptive and regression-based analyses, exploratory computational approaches were applied for internal signal detection and feature attribution. These analyses were not intended for predictive model development or clinical implementation. Binary classification trees (CART), logistic regression–based ROC analysis, permutation importance, and SHAP-based attribution were used to examine recurrence-associated patterns related to margin width and radiotherapy.

The registry also contained follow-up–related variables, including interval-coded recurrence-free and survival fields (e.g. RFS-type variables across predefined time windows). However, these variables did not provide a sufficiently uniform patient-level time-to-event structure for fully standardized reconstruction of formal Kaplan–Meier or Cox models within the present revision workflow. Accordingly, survival-oriented findings were treated as supportive rather than primary inferential evidence and interpreted cautiously.

## Results

### Patient characteristics and treatment patterns

**Fig. 1:**
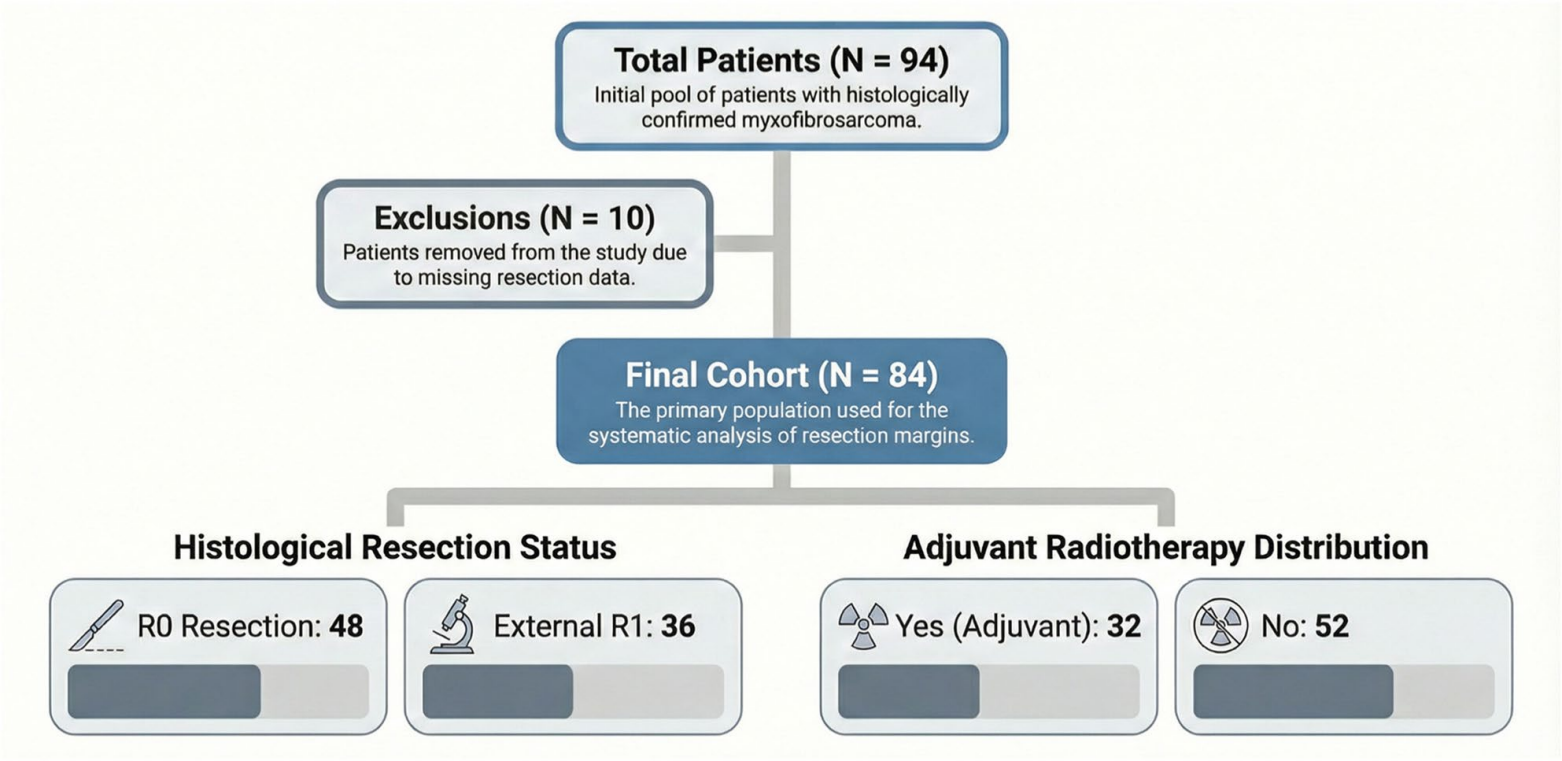
Study cohort and clinical subgroups. Flowchart of patient selection from 2000 to 2023. Of 94 patients with histologically confirmed myxofibrosarcoma, 10 were excluded due to missing resection margin data. The final cohort of 84 patients is stratified by histological resection status (R0 vs. external R1) and radiotherapy status. Note: the high R1 proportion (43%) reflects the institution’s role as a tertiary referral center receiving patients after external primary surgery

A total of 94 patients with histologically confirmed myxofibrosarcoma were identified between 2000 and 2023. Ten patients were excluded because of missing resection data, resulting in a final analytic cohort of 84 patients. Among these, 48 patients (57%) underwent R0 resection, whereas 36 patients (42%) had an externally determined R1 resection status. All patients were treated with the intention of achieving R0 resection. Adjuvant radiotherapy was administered in 32 cases (38%), while 52 patients (61%) did not receive adjuvant radiotherapy (Fig. 1).

The mean age at the time of surgery was 65.7 years (SD 15.5), and the mean follow-up duration was 5.3 years (SD 2.9). The cohort comprised 38 male patients (45%) and 46 female patients (55%). Tumor grade was G1 in 18 patients (21%), G2 in 28 patients (33%), and G3 in 33 patients (40%); grade was undocumented in 5 patients (6%). Median tumor size was 6.6 cm (2.4–10.9). Tumors were located in the lower extremity in 46 patients (55%), in the upper extremity in 24 patients (29%), and at other sites in 14 patients (16%). Referral for external re-resection was documented in 47 patients, of whom 36 (77%) had undergone prior external surgery and 11 (23%) had not; referral status was undocumented in 37 patients (44%). Local recurrence occurred in 28 of 79 patients with documented recurrence status (35%), while recurrence status was missing in 5 cases. Baseline characteristics are summarized in Table 1.

**Table 1:** Characteristics of the patient cohort

Age at Operation	65.7 years (SD 15.5)
Follow-Up Period	5.3 years (SD 2.9)
Sex	
Male	38 (45%)
Female	46 (55%)
Tumor Grade	
G1	18 (21%)
G2	28 (33%)
G3	33 (40%)
Undocumented	5 (6%)
Tumor Size	6.6 cm (2.4–10.9)
Depth	
Subfascial	35 (42%)
Epifascial/Subcutaneous	24 (28%)
Missing Value	25 (30%)
Location	
Lower Extremity	46 (55%)
Upper Extremity	24 (29%)
Other	14 (16%)
Referral for external re-resection	
Yes	36 (43%)
No	11 (13%)
Undocumented	37 (44%)
Local Recurrence	28/79 (35%); 5 missing

### Impact of resection margin width on local recurrence

Numeric margin values were documented in 38/84 patients (45.2%), with a median of 0.1 mm (IQR 0.1–0.575; range 0.1–8.0). Twenty of 38 documented values were exactly 0.1 mm, likely reflecting a reporting convention rather than precise micrometry. Margin distribution: <1 mm (*n* = 32, 38.1%), 1–5 mm (*n* = 5, 6.0%), > 5 mm (*n* = 1, 1.2%), missing (*n* = 46, 54.8%) due to external resection.

**Fig. 2:**
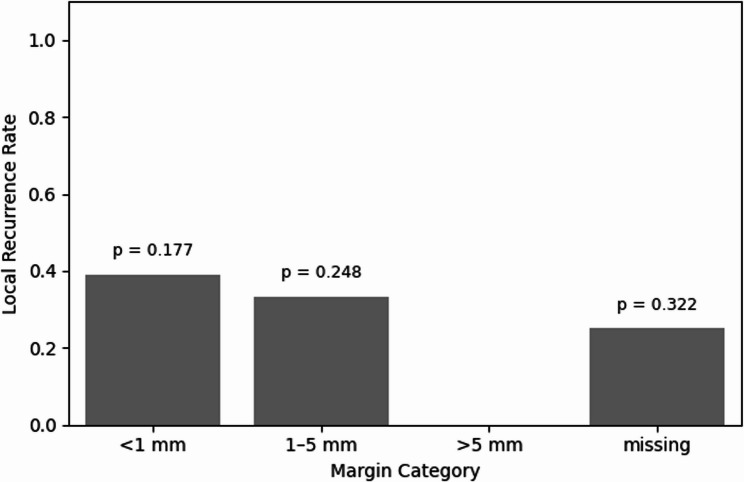
Local recurrence rates by margin categories. Bar plot showing local recurrence rates stratified by resection margin categories: <1 mm, 1–5 mm, > 5 mm, and missing. Statistical comparisons were performed using the Chi-squared test or Fisher’s exact test, as appropriate. No significant differences were observed among groups (all *p* > 0.1)

When stratified by categorical margin width (< 1 mm, 1–5 mm, > 5 mm), the local recurrence rates were 38%, 33%, and 0%, respectively, with 25% among cases with missing data (Fig. 2). Although a trend toward reduced recurrence with increasing margin width was observed, no statistically significant differences were detected between groups (Chi-squared test: *p* = 0.177, *p* = 0.248, *p* = 0.322, respectively).

**Fig. 3:**
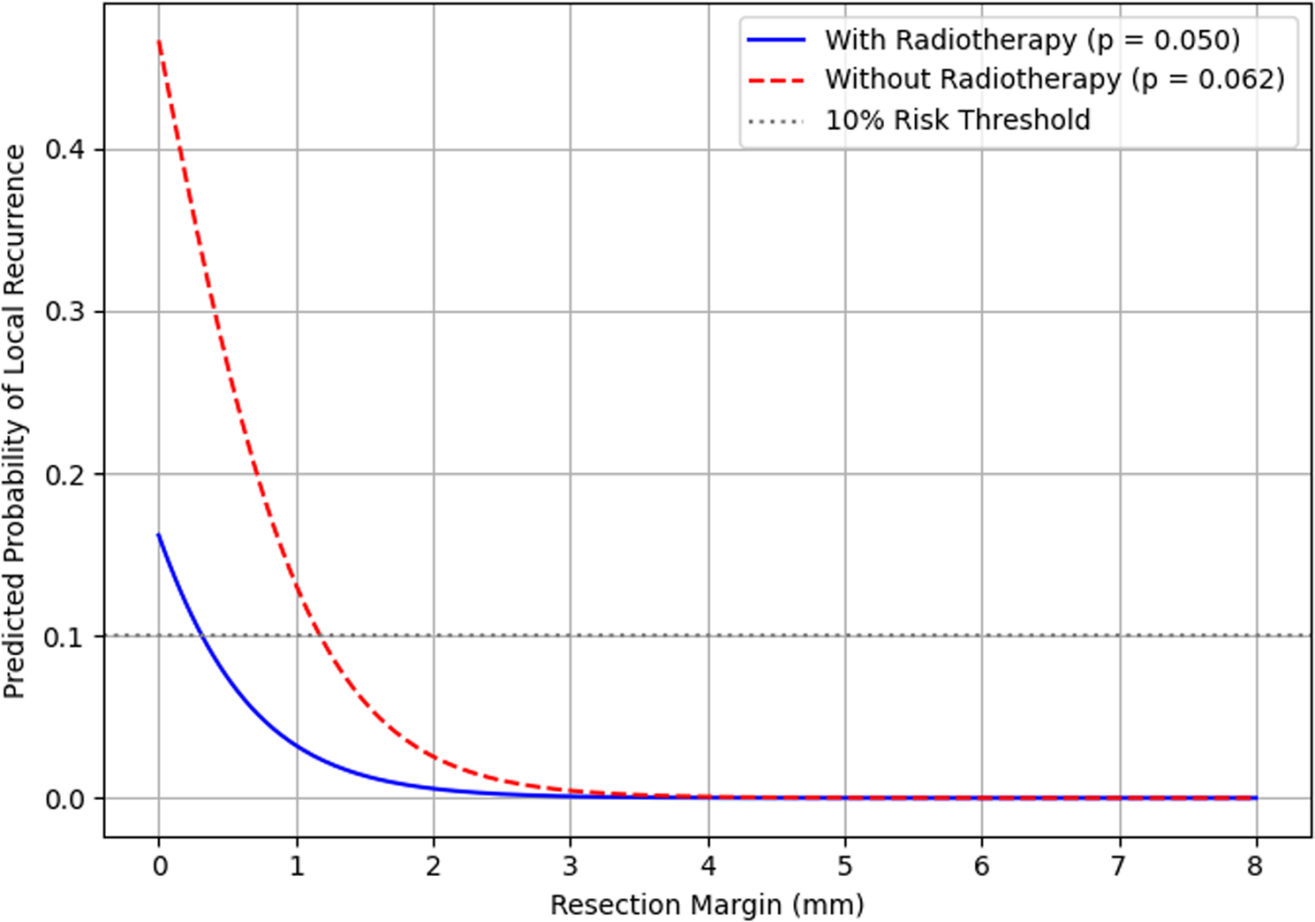
Exploratory logistic regression visualization of recurrence probability vs. resection margin. Predicted local recurrence probability as a function of margin width (mm), stratified by radiotherapy status, based on an exploratory logistic regression model fitted to the subcohort with documented numeric margins (*n* = 38). This visualization illustrates the general directional trend; specific numerical thresholds should not be read from the curves given the small sample, wide confidence intervals, and exploratory nature of the analysis. The stratified tabular data in Table 2 provide the primary descriptive evidence. A supportive univariable Cox proportional hazards analysis yielded HR = 0.85 per mm (95% CI 0.70–1.02, *p* = 0.073); however, fully standardized re-estimation of time-to-event models was not feasible with the available registry variables. All findings should be interpreted as exploratory

A logistic regression model suggested a continuous inverse relationship between resection margin width and local recurrence probability, with lower predicted recurrence in the radiotherapy subgroup across the observed margin range (Fig. 3). However, confidence intervals were wide, the subgroup with documented numeric margins was small (*n* = 38), and precise numerical thresholds should not be derived from this exploratory visualization. The stratified tabular analyses (Table 2) provide the primary descriptive evidence for margin- and radiotherapy-associated recurrence patterns. In a supportive analysis, Cox proportional hazards modeling yielded a univariable HR of 0.85 per mm (95% CI: 0.70–1.02, *p* = 0.073), although a fully standardized re-estimation of survival-type models was not feasible with the available follow-up variables.

### Stratified local recurrence by resection margin and radiotherapy status

Within the < 1 mm margin subgroup, local recurrence was observed in 30.0% (6/20) of patients who received radiotherapy, compared with 50.0% (6/12) of those who did not (Fisher’s exact *p* = 0.288). Across the remaining margin strata, recurrence rates also varied by radiotherapy exposure, although no statistically significant association was identified. Owing to small subgroup sizes, low event counts, and likely confounding by indication, these analyses should be regarded as descriptive rather than inferential (Table 2).

**Table 2:** Cross-tabulation of local recurrence according to resection margin category and radiotherapy exposure (complete-case cohort, *n* = 79)

Margin	RT	*n*	LR *n*	LR %
< 1 mm	Yes	20	6	30.0
< 1 mm	No	12	6	50.0
1–5 mm	Yes	2	1	50.0
1–5 mm	No	3	0	0.0
> 5 mm	No	1	0	0.0
Missing	Yes	10	1	10.0
Missing	No	31	14	45.2
Total		79	28	35.4

Cross-tabulation of local recurrence according to resection margin category and radiotherapy exposure in the complete-case cohort. Five patients with missing local recurrence status were excluded. Within the < 1 mm subgroup, local recurrence occurred in 30.0% (6/20) of patients receiving radiotherapy versus 50.0% (6/12) of those without radiotherapy (Fisher’s exact *p* = 0.288; not significant)

### Effect of adjuvant radiotherapy

**Fig. 4:**
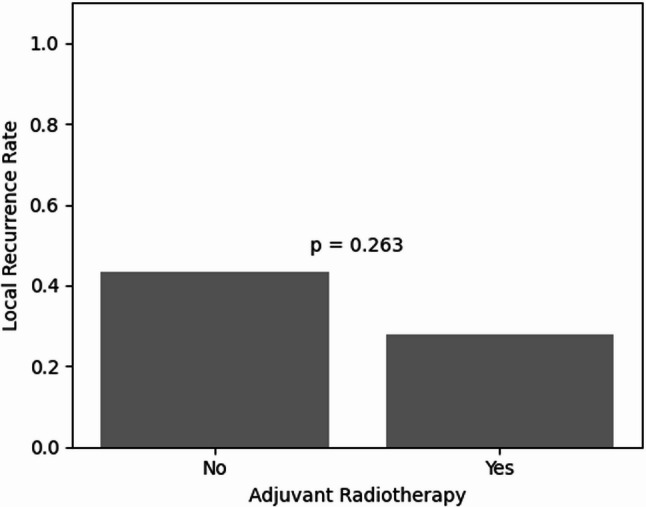
Local recurrence rates by adjuvant radiotherapy. Descriptive bar plot of local recurrence rates stratified by radiotherapy exposure in the complete-case cohort. Recurrence was numerically lower in irradiated patients, although no statistically significant difference was observed. In the < 1 mm margin subgroup, local recurrence occurred in 30.0% (6/20) with radiotherapy and 50.0% (6/12) without radiotherapy (Fisher’s exact *p* = 0.288). These subgroup findings should be interpreted cautiously because of small sample size, sparse events, and confounding by indication. In exploratory multivariable Cox regression, radiotherapy was retained after backward stepwise selection but remained non-significant (adjusted HR 0.52, 95% CI 0.18–1.46; *p* = 0.212)

Radiotherapy was associated with a numerically lower recurrence rate (27% vs. 43%) but this did not reach significance (*p* = 0.263) (Fig. 4). In multivariable Cox regression, RT was retained after backward stepwise selection but remained non-significant (adjusted HR 0.52, 95% CI: 0.18–1.46, *p* = 0.212). These associations must be interpreted with caution given confounding by indication: RT decisions are risk-adapted, and recipients likely had higher baseline risk. Neoadjuvant versus adjuvant timing could not be distinguished.

### Grade-stratified recurrence

Grade-stratified recurrence rates were similar in this cohort, with local recurrence observed in 17/46 low-grade tumors (37.0%) and 11/33 high-grade tumors (33.3%). Within the < 1 mm subgroup, recurrence occurred in 7/18 low-grade and 5/14 high-grade cases. These subgroup findings should not be overinterpreted, as treatment selection, limited sample size, and residual confounding likely influenced the observed patterns.

### Machine learning-derived margin thresholds and predictive modeling

Eight candidate cutoffs met the minimum group size requirement (see Table 3).

**Table 3:** Descriptive exploratory analysis of candidate resection margin thresholds and local recurrence with multiple-testing correction

Cutoff	*n*<	*n*≥	LR<	LR≥	*p* unadj	*p* Bonf	*p* FWER
0.2	20	18	10	3	0.043	0.347	0.110
0.3	24	14	12	1	0.012	0.094	0.028
0.4	26	12	12	1	0.030	0.240	0.064
0.5	27	11	12	1	0.060	0.478	0.119
0.6	28	10	12	1	0.118	0.946	0.253
0.7	29	9	12	1	0.126	1.000	0.270
0.8	30	8	12	1	0.222	1.000	0.447
1.0	32	6	12	1	0.643	1.000	0.938

The 0.3 mm cutoff yielded the smallest unadjusted p-value. Permutation FWER (*p* = 0.028) was favorable; Bonferroni (*p* = 0.094) did not reach conventional significance. The permutation FWER is the more appropriate correction for correlated tests; both are reported for transparency. This threshold primarily distinguishes a measurement-floor cluster (0.1 mm, *n* = 20) from more widely excised specimens.

In a multivariable logistic regression model restricted to cases with documented margin width (*n* = 38), a margin ≥ 0.3 mm was associated with lower odds of local recurrence (OR = 0.086, 95% CI 0.008–0.968; *p* = 0.047). However, this analysis was severely underpowered (13 events across 5 covariates), and bootstrap resampling (2,000 iterations) demonstrated marked instability with wide dispersion of effect estimates (97.5th percentile OR ≈ 1.17). This result must be interpreted as exploratory.

### Exploratory margin thresholds and feature attribution

**Fig. 5:**
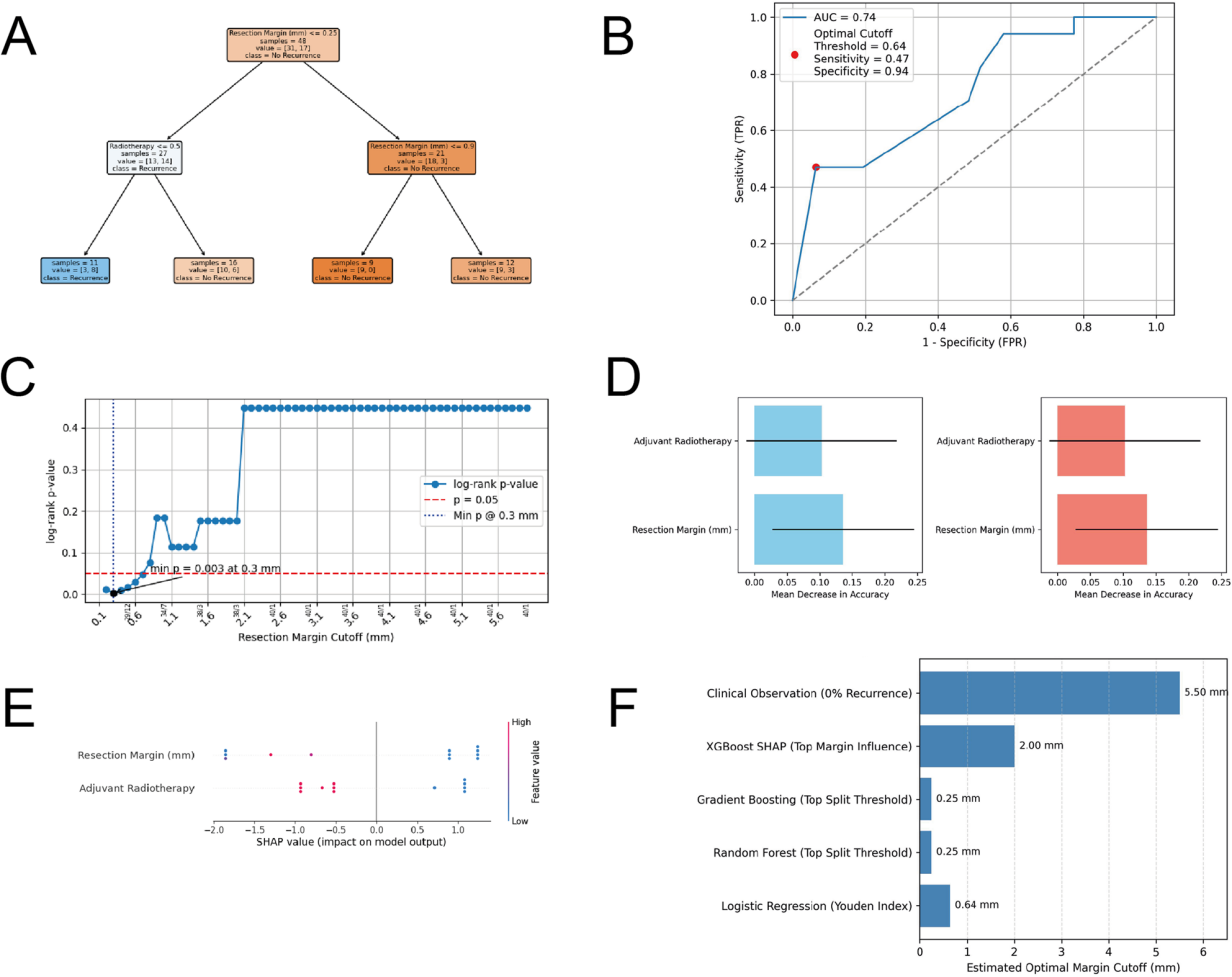
Exploratory Computational Analyses of Resection Margin Signals.** A** Decision tree model based on resection margin width and radiotherapy status illustrating data-driven partitioning of recurrence patterns. Radiotherapy and a margin threshold near 0.25 mm emerged as principal splits; findings are exploratory and sample-dependent. **B** Receiver operating characteristic curve derived from logistic regression including margin width and radiotherapy. The model yielded an area under the curve (AUC) of 0.74. The Youden-optimal threshold was 0.64 mm (sensitivity 0.47; specificity 0.94), indicating high specificity but limited sensitivity in this cohort. **C** Exploratory threshold scan across continuous margin cutoffs (0.1–6.0 mm). The smallest unadjusted p-value occurred near 0.3 mm (*p* = 0.003); however, limited sample size and multiplicity preclude confirmatory inference. **D **Permutation feature-importance analyses from random forest (left) and gradient boosting (right) showing the relative contribution of margin width and radiotherapy to model performance. **E** SHAP summary plot illustrating feature influence and directionality in an XGBoost model; smaller margins and absence of radiotherapy were associated with higher predicted recurrence risk. **F** Comparison of candidate margin thresholds across analytic approaches, demonstrating partially overlapping estimates ranging from submillimeter values to wider excision ranges. These exploratory results should be interpreted as internal signal detection within a limited cohort and not as evidence for a clinically validated or surgically targetable cutoff

To further investigate potential margin-related signals, we applied complementary computational approaches (Fig. 5A–F), explicitly framed as exploratory. A decision tree model identified radiotherapy status and a margin threshold near 0.25 mm as the principal data-driven splits associated with recurrence patterns (Fig. 5A). Receiver operating characteristic analysis using logistic regression yielded an area under the curve of 0.74, with a Youden-optimal cutoff at 0.64 mm (sensitivity 0.47; specificity 0.94; Fig. 5B), indicating high specificity but limited sensitivity.

Independent threshold scanning across continuous margin values showed the lowest unadjusted p-value at approximately 0.3 mm (*p* = 0.003; Fig. 5C), consistent with the logistic threshold analysis, though multiplicity and limited sample size preclude confirmatory interpretation. Ensemble feature-ranking methods (random forest and gradient boosting) consistently identified margin width and radiotherapy as the highest-ranking variables (Fig. 5D). SHAP analysis further quantified variable influence and demonstrated that smaller margins and absence of radiotherapy were directionally associated with higher predicted recurrence risk (Fig. 5E).

Across computational approaches, convergence analysis showed partially overlapping candidate thresholds ranging from 0.25 to 2.0 mm (Fig. 5F). Given sparse events, measurement-floor clustering, and lack of external validation, these thresholds should be considered hypothesis-generating rather than clinically prescriptive. Collectively, the analyses suggest a potential signal within the submillimeter range that warrants prospective validation, while reinforcing that margin status (R0, “tumor not on ink”) and anatomical barrier quality remain the primary determinants of surgical adequacy.

Across multiple analytical approaches, including ROC analysis, threshold scanning, decision-tree partitioning, and SHAP-based feature attribution, resection margin width consistently emerged as a prominent predictor associated with local recurrence. Exploratory threshold analyses identified the smallest unadjusted signal near 0.3 mm, although limited sample size and multiplicity preclude confirmatory interpretation. Convergence across computational methods suggested partially overlapping candidate ranges spanning submillimeter to wider excision margins (Fig. 5F), reflecting methodological variability rather than a single definitive cutoff. Collectively, these findings indicate a potential signal within the very-close-margin range that warrants prospective validation, while reinforcing that surgical adequacy remains primarily defined by margin status (R0, “tumor not on ink”) and anatomical barrier context rather than a fixed numeric threshold.

## Discussion

In this retrospective single-center cohort, we observed an exploratory signal suggesting that very close negative margins may be associated with increased local recurrence risk in myxofibrosarcoma. Within the subgroup with documented millimeter margins, the strongest unadjusted separation was seen near 0.3 mm. However, given the limited sample size, sparse events, and multiplicity of testing, this finding should be regarded as hypothesis-generating rather than confirmatory. Although complementary computational approaches produced partially overlapping signals within the submillimeter to low-millimeter range, these analyses were derived from the same dataset and do not constitute independent validation. Although interval-coded follow-up variables were available in the registry, they did not support a fully harmonized re-estimation of patient-level time-to-event models in the present revision, so survival-oriented findings remain supportive rather than definitive.

Instead, our findings support the concept that margin status remains primary, whereas margin width is a secondary descriptor whose prognostic relevance depends on anatomical barrier quality. A very close negative margin through fascia may not carry the same biological meaning as the same measured distance through infiltrated subcutaneous tissue. This distinction could not be addressed in our registry and represents a major limitation. In addition, submillimeter measurements on formalin-fixed, inked specimens are inherently imprecise because of shrinkage and processing artefacts, and infiltrative extensions were not systematically mapped. Accordingly, the observed 0.3 mm signal is best understood as a possible marker of a very-close-margin zone that requires prospective validation rather than as a definitive cutoff for surgical decision-making.

In the broader soft tissue sarcoma literature, there remains no consensus on a “safe” margin width. Historical guidelines have varied substantially: the Association of Directors of Anatomic and Surgical Pathology (1999) recommended ≥ 2 cm, while the 2010 ESMO guidelines proposed ≥ 1 cm, acknowledging that narrower margins may suffice in anatomically constrained sites [9, 13, 14]. More recent studies suggest that histologic subtype should guide margin targets. For example, Wittenberg et al. found that low-grade STS benefits from margins ≥ 2 mm, while high-grade sarcomas showed no significant detriment with narrower R0 margins when treated with adjuvant therapy [15]. Several large series indicate that recurrence rates plateau beyond 5 mm, suggesting that margins > 1 cm may offer only marginal oncologic benefit [16]. Our results extend this paradigm by reinforcing that a strictly negative margin, rather than its absolute width, is the most critical determinant of local control, particularly in MFS.

The role of adjuvant radiotherapy in cases with close but negative margins is increasingly recognized. Both ESMO and NCCN recommend adjuvant radiotherapy for large, deep, or high-grade tumors, especially when margin width is limited. Recent evidence supports “planned close margin” strategies in anatomically critical locations, provided adjuvant radiotherapy is employed [10]. In our study, adjuvant radiotherapy showed a trend toward improved local control, although it did not reach statistical significance, possibly due to sample size. Nonetheless, the directionality aligns with reports from Boughzala-Bennadji et al. and others demonstrating significant benefit from radiotherapy in MFS, particularly when surgical margins are narrow [7]. Interpretation of radiotherapy effects is further complicated by confounding by indication. Adjuvant treatment decisions were risk-adapted and influenced by tumor size, depth, grade, anatomical constraints, and evolving institutional protocols. Neo- and adjuvant radiotherapy could not be reliably separated in the registry variables, and treatment allocation was not randomized. Consequently, observed associations between radiotherapy and recurrence outcomes should be interpreted as descriptive rather than causal. The potential role of neoadjuvant chemoradiotherapy cannot be reliably assessed in the present cohort. In contemporary sarcoma practice, preoperative radiotherapy may be considered for selected large, deep, or anatomically constrained tumors to facilitate local control while preserving function; however, the available registry variables did not permit sufficiently robust separation of neoadjuvant from adjuvant treatment pathways for meaningful subgroup analysis.

As expected, R1 status remained a strong negative prognostic factor. Patients with positive microscopic margins had significantly higher local recurrence rates. This is consistent with prior studies across STS subtypes, reinforcing that true margin negativity - regardless of absolute millimeter measurement - is vital for durable local control.

Across multiple exploratory analytical approaches, resection margin width repeatedly emerged as a relevant variable associated with local recurrence. However, the apparent convergence of signals near 0.3 mm should not be interpreted as independent confirmation of a clinically established threshold, since all analyses were derived from the same limited dataset and were affected by sparse events, measurement-floor clustering, and model instability. Rather than defining a surgically actionable cutoff, these findings suggest the possibility of a “very close margin zone” in which recurrence risk may remain elevated despite R0 status. The clinical implication is therefore not to aim for a specific submillimeter target, but to interpret very close pathological margins in the broader context of anatomical barriers, radiotherapy, and surveillance strategy.

This study has several limitations. As a retrospective, single-center analysis, it is inherently subject to selection bias and potential confounding. Furthermore, the cohort reflects the case mix of a tertiary referral center over a prolonged study period. A substantial proportion of patients underwent prior external surgery or re-resection, contributing to the relatively high R1 rate and limiting comparability with prospective sarcoma center benchmarks. Over the 23-year inclusion period, surgical techniques, imaging standards, and multidisciplinary treatment strategies evolved considerably, which may have introduced temporal heterogeneity. These structural factors restrict external validity and preclude generalization of any numeric margin signal to contemporary standardized sarcoma care pathways. The relatively small number of patients within individual sarcoma subtypes, especially for rare histologies, limits the power to draw definitive conclusions regarding subtype-specific margin thresholds. While machine learning techniques enhance interpretability and robustness, their findings require prospective validation before clinical implementation. Machine learning analyses were conducted on a small dataset with limited events and therefore carry a substantial risk of overfitting. Although internal validation techniques such as cross-validation and bootstrap resampling were applied, these approaches cannot substitute for external validation. Accordingly, computational findings should be interpreted as exploratory pattern recognition rather than predictive modeling suitable for clinical deployment.

Additionally, variations in adjuvant treatment protocols, radiotherapy delivery, and pathological assessment of margins over the study period may have introduced heterogeneity. A further limitation is that follow-up information was captured primarily through interval-coded recurrence-free and survival variables rather than a fully harmonized event-time dataset, restricting the reproducibility of standardized time-to-event reanalysis within the present revision. Sensitivity analyses excluding cases flagged as external surgery or re-resection were directionally consistent but remained too sparse for robust inference, underscoring that the present study should be interpreted as a case-mix-aware exploratory analysis rather than a definitive margin study in a primary resection cohort. Despite these constraints, the convergence of findings across multiple analytical approaches strengthens the validity of our results.

## Conclusion

Our findings support the prognostic importance of achieving a histologically negative margin in myxofibrosarcoma, while suggesting that very close negative margins may still define a higher-risk zone for local recurrence. In this exploratory analysis, the signal observed near 0.3 mm should be interpreted as hypothesis-generating rather than practice-informing. Surgical adequacy in MFS remains primarily determined by R0 status, anatomical barrier quality, and multidisciplinary treatment context rather than by a fixed submillimeter cutoff. Prospective multicenter studies with standardized pathology and harmonized follow-up/event-time data are required before any quantitative threshold can be considered clinically actionable.

## Data Availability

The datasets generated and/or analyzed during the current study are not publicly available due to institutional data protection regulations and patient privacy concerns but are available from the corresponding author on reasonable request.
